# Highly concentrated collagen/chondroitin sulfate scaffold with platelet-rich plasma promotes bone-exposed wound healing in porcine

**DOI:** 10.3389/fbioe.2024.1441053

**Published:** 2024-09-24

**Authors:** Zhihao Li, Qian Li, Akhlaq Ahmad, Zhongjie Yue, Hongxia Wang, Guofeng Wu

**Affiliations:** ^1^ Department of Spinal Surgery, Jingzhou Hospital Affiliated to Yangtze University, Jingzhou, Hubei, China; ^2^ Medical Laboratory of Jingzhou Hospital Affiliated to Yangtze University, Jingzhou, Hubei, China; ^3^ Institutes of Integrative Medicine, Fudan University, Shanghai, China; ^4^ Department of Orthopedics, Southern University of Science and Technology Hospital, Shenzhen, Guangdong, China

**Keywords:** bone-exposed wounds, high concentration collagen, platelet-rich plasma, vascular regeneration, mechanical strength

## Abstract

In the case of wounds with exposed bone, it is essential to provide not only scaffolds with sufficient mechanical strength for protection, but also environments that are conducive to the regeneration of tissues and blood vessels. Despite the excellent biocompatibility and biodegradability of collagen and chondroitin sulfate, they display poor mechanical strength and rapid degradation rates. In contrast to previous methodologies that augmented the mechanical properties of biomaterials through the incorporation of additional substances, this investigation exclusively enhanced the mechanical strength of collagen/chondroitin sulfate scaffolds by modulating collagen concentrations. Furthermore, platelet-rich plasma (PRP) was employed to establish optimal conditions for vascular and tissue regeneration at the wound site. High-concentration collagen/chondroitin sulfate (H C-S) scaffolds were synthesized using high-speed centrifugation and combined with PRP, and their effects on endothelial cell proliferation were assessed. A porcine model of bone-exposed wounds was developed to investigate the healing effects and mechanisms. The experimental results indicated that scaffolds with increased collagen concentration significantly enhanced both tensile and compressive moduli. The combination of H C-S scaffolds with PRP markedly promoted endothelial cell proliferation. *In vivo* experiments demonstrated that this combination significantly accelerated the healing of porcine bone-exposed wounds and promoted vascular regeneration. This represents a promising strategy for promoting tissue regeneration that is worthy of further exploration and clinical application.

## 1 Introduction

The skin functions as a barrier between the internal and external environments of the human body, representing the largest organ in the body and performing a multitude of vital functions for survival (Haxaire et al., 2023). The presence of stem cells enables the injured epidermis to stimulate self-regeneration. However, in cases of deep injuries, such as exposed bones, the healing process is often insufficient, leading to chronic wounds (Pena and Martin, 2024). Severe skin injuries frequently require autologous skin grafting treatments (Kahn et al., 2024). Nonetheless, the availability of autologous healthy skin tissue for grafts is limited. The use of allogeneic skin tissue does provide an alternative, but it also carries risks of infection and immune rejection (Schlottmann et al., 2024).

Tissue engineering materials have demonstrated considerable potential as a means of facilitating the regeneration of wound-healing skin tissues (Wang et al., 2023; Cao et al., 2023). In recent decades, a variety of multifunctional biomaterials have been developed with the capacity to provide barrier protection to damaged tissues, reduce wound pain, and facilitate healing through tissue regeneration (Motsoene et al., 2023; Guo et al., 2024). The role of collagen and chondroitin sulfate in promoting wound healing has been the subject of extensive study (Rong et al., 2023). A sponge-like wound dressing, comprising gelatin, chondroitin sulfate, and collagen, has been developed by researchers and has been demonstrated to facilitate wound healing (Wang et al., 2006). However, scaffolds made from collagen and chondroitin sulfate at conventional concentrations are susceptible to disintegration upon contact with water and exhibit poor mechanical properties. Liang et al. (2010) reported that collagen protein can be concentrated via centrifugation, resulting in the subsequent production of high concentration collagen-chondroitin sulfate (H C-S) scaffolds with significantly enhanced mechanical properties and water absorption.

The absence of subcutaneous fat, muscle tissue, and slow vascularisation are regarded as the primary characteristics of bone-exposed wounds (Darwish et al., 2024). In addition to providing a physical barrier, facilitating vascular and tissue regeneration is of critical importance for the process of wound healing (Fani et al., 2024). Researchers have endeavoured to enhance the properties of biomaterials in order to facilitate vascular and tissue regeneration (Song et al., 2024). Among the strategies that have been considered, the addition of platelet-rich plasma (PRP) has been identified as a promising therapeutic approach. PRP is obtained by centrifuging animal or human whole blood to produce a high concentration of platelet plasma containing multiple growth factors (GFs), including platelet-derived growth factor (PDGF), transforming growth factor-β (TGF-β), insulin-like growth factor (IGF), epidermal growth factor (EGF), and vascular endothelial growth factor (VEGF) (Pixley et al., 2023). The GFs present in PRP have been demonstrated to facilitate a number of essential processes in wound healing, including cell attachment, migration, differentiation, and extracellular matrix synthesis. Of particular importance is their ability to promote vascular regeneration (Pineda-Cortel et al., 2023). The combination of PRP and biomaterials in tissue engineering provides mechanical support and enables the sustained local release of bioactive molecules at the site of injury, making it a low-cost and efficient potential treatment method (Grzelak et al., 2024; Wu et al., 2022).

**FIGURE 1:**
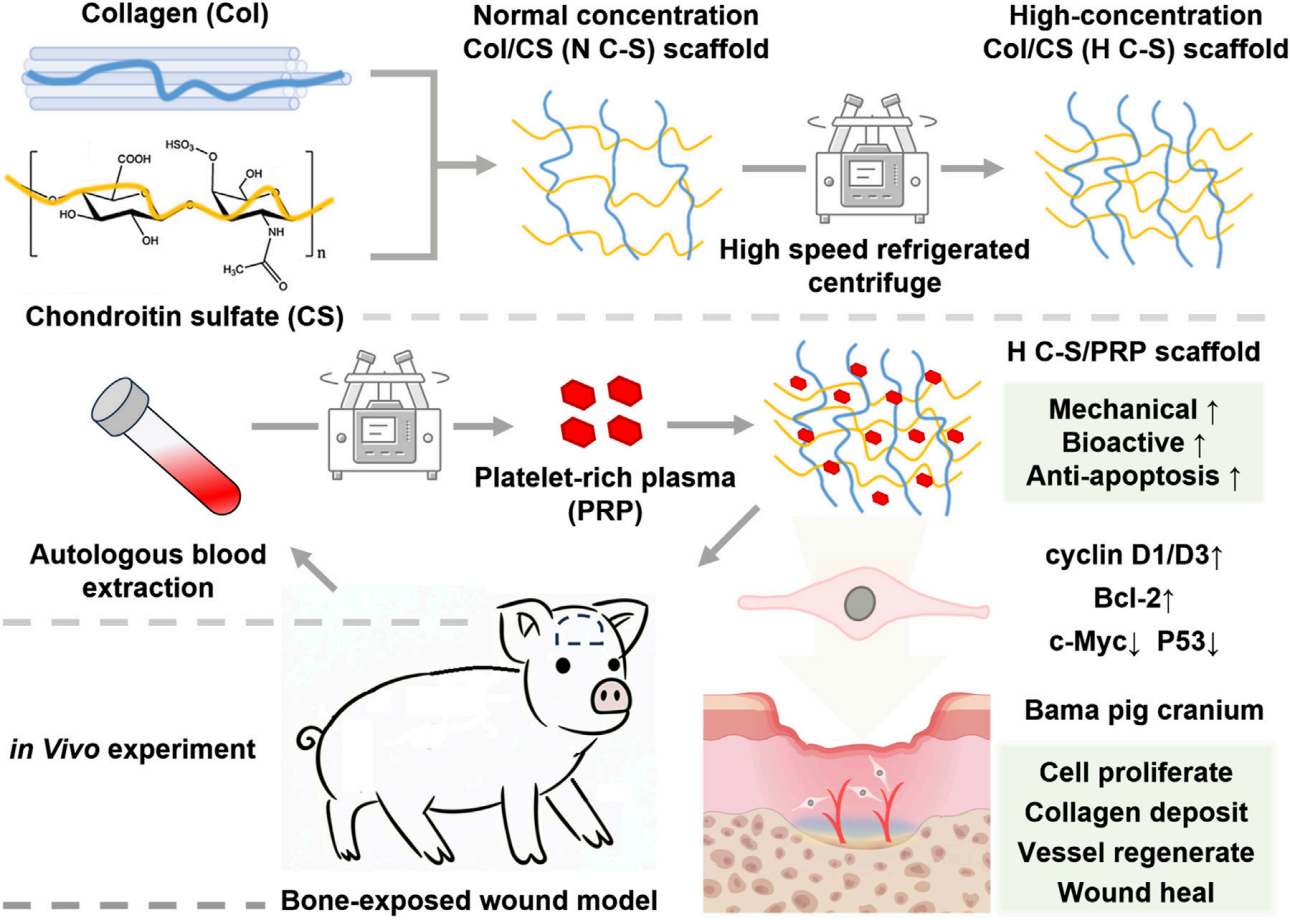
The schematic illustration of H C-S/PRP scaffold that promotes bone-exposed wound healing in porcine.

Collagen and chondroitin sulfate are components of the extracellular matrix, renowned for their exemplary biocompatibility and biodegradability. However, due to their poor mechanical properties, they often require combination with other materials to enhance their mechanical strength, which can result in a reduction in biodegradability and biocompatibility of the scaffold materials. In this experiment, the mechanical strength of the scaffold was enhanced without the addition of other materials through a simple adjustment of the collagen concentration. The scaffold was used in conjunction with PRP to repair bone-exposed wounds in a porcine model. Initially, H C-S scaffolds were synthesized and their mechanical strength was evaluated. Subsequently, the composite material was combined with PRP and its effects on human umbilical vein endothelial cells (HUVECs) were tested *in vitro*. Finally, this composite material was applied to bone-exposed wounds in porcine, and the healing effects at different time points were monitored, along with histological staining to elucidate the potential mechanisms by which the composite material promoted healing of the bone-exposed wounds. The schematic illustration is presented in Figure 1.

## 2 Materials and methods

### 2.1 Materials

The type I collagen (Rat Tail, purity >90%) was purchased from Guangzhou Zuo Ke Biotechnology Development Co., Ltd. Chondroitin sulfate A (molecular weight 18 kDa, purity 98%) was purchased from Guangzhou Zuo Ke Biotechnology Development Co., Ltd. Acetic acid was purchased from Shanghai Maclin Biochemical Technology Co., Ltd. HUVECs were purchased from Guangzhou Yongjin Biotechnology Co., Ltd. Experimental Bama pigs were purchased from Jiangsu Ailingfei Biotechnology Co., Ltd. Deionized water (>18.2 MΩcm) was used when water was involved.

### 2.2 Fabrication of H C-S scaffolds

A method previously described in the literature (Liang et al., 2010) was employed to successfully produce H C-S scaffolds. The process commenced with the dissolution of 110 mg of type I collagen in 40 mL of 0.5 M acetic acid. The solution was then subjected to vigorous mixing at 13,500 rpm for 20 min in an ice-water cooled container. Subsequently, 11 mg of chondroitin sulfate was dissolved in 2 mL of 0.5 M acetic acid, thoroughly mixed and gradually added to the collagen solution. Following thorough mixing, the combined mixture was lyophilized, resulting in a normal concentration collagen/chondroitin sulphate (N C-S) scaffold. The concentrations of collagen and chondroitin sulfate in the N C-S solution are 2.6 g/L and 0.26 g/L, respectively. In order to obtain a higher concentration scaffold, the N C-S solution was subjected to centrifugation at 38,720 g for a period of 20 min, utilising a high-speed refrigerated centrifuge (Avanti JXN-26). Following the centrifugation process, the supernatant, which constituted 80% of the total solution, was discarded, and the remaining solution was thoroughly mixed with the collagen precipitate. This final mixture was then lyophilised, resulting in the production of an H C-S scaffold. The concentrations of collagen and chondroitin sulphate in the H C-S solution after concentration treatment are approximately 11.13 g/L and 0.26 g/L respectively.

### 2.3 Testing of water absorption and mechanical properties of H C-S scaffolds

The fabricated H C-S and N C-S scaffolds were cut into discs of 10 mm diameter, dried, and their weights were recorded. Subsequently, the scaffolds were immersed in culture medium for 24 h to achieve complete hydration. Thereafter, the weights of the water-saturated scaffolds were determined (n = 6). Moreover, the scaffolds were shaped into rectangular specimens with dimensions of 22 mm in length, 5 mm in width, and 3 mm in height. The specimens were subjected to tensile testing using an electronic universal testing machine (Instron model 5,565) at a strain rate of 1.4 mm/min. The tensile modulus was calculated by fitting the experimental stress (σ) and strain (ε) data according to the manufacturer’s instructions. Similarly, the compressive modulus of each 10 mm diameter disc was measured using the same testing machine. Each disc was compressed at a strain rate of 5% s^−1^ for 3 s, and the stress-strain curves were recorded by a computer data acquisition system to derive the average compressive modulus. The weight of each group of scaffolds was recorded, after which they were placed in 100 mL of saline solution and subjected to cyclic shaking at 100 rpm at a temperature of 37°C ± 0.5°C. The fluid was replaced on a weekly basis, and following 10, 20 and 30 days, three samples from each group were taken, dried and weighed. In the material degradation tests, the weighed samples were subjected to an ultimate stress test. The results are presented in Supplementary Figure S1.

### 2.4 PRP specimens were obtained and identified

All experimental procedures were conducted in strict accordance with the guidelines set forth by the Animal Care and Ethics Committee and were approved by the Ethics Committee of the Medical Department of Yangtze University. The isolation and extraction of PRP samples were conducted in accordance with the methodology previously outlined in the literature (Everts et al., 2021). Blood samples were obtained from Bama miniature pigs. In summary, the pigs were anaesthetised with isoflurane, and a venous transfusion needle was inserted into the posterior lateral saphenous vein of the hind limb. Subsequently, a 20-mL sample of fresh blood was extracted using a syringe. The sample was subjected to centrifugation at 300 g for a period of 10 min, with the objective of removing the red and white blood cells. The resulting supernatant and intermediate layers were then collected. The collected solution was then subjected to a further centrifugation at 1,200 g for 15 min at 4°C, resulting in the isolation of approximately 2 mL of PRP from the bottom fraction. The platelet concentrations in both the extracted platelet-rich plasma and whole blood were quantified using a hemocytometer (Acmec, Shanghai, China). The PRP supernatant was prepared by activation with 100 µL of 10% calcium chloride (Aladdin). ELISA kits (Abbkine) were employed to quantify the levels of TGF-β, PDGF and VEGF in the PRP supernatant. According to the classification system proposed by Harrison et al. (2018), the prepared PRP is categorized as PRP IIA1.

### 2.5 Fabrication and electron microscope scanning of H C-S/PRP

The prepared H C-S was trimmed to a size of 5 mm × 5 mm, and 2 mL (×1) PRP IIA1 was added to each sample. Subsequently, the samples were incubated in 5 mL EP tubes for 30 min, resulting in the formation of the H C-S/PRP scaffolds. The scaffolds were subsequently stored overnight in a freezer set to −80°C and then freeze-dried in a freeze dryer (Lichen Technology, China). Following the freeze-drying process, a specimen holder comprising conductive tape was prepared, and the specimens were attached to the tape with great care. In order to enhance the conductivity of the samples, a thin layer of platinum was applied to their surfaces via spraying. The platinum-sprayed samples were subsequently subjected to examination in a scanning electron microscope (ZEISS Merlin).

### 2.6 Detection of slow release of GFs in H C-S/PRP

The prepared H C-S/PRP was placed in 10 mL of physiological saline and subsequently transferred to a constant temperature incubator maintained at 37°C. On days 0.5, 2, 5, 10, 15 and 22 of cultivation, the supernatant was collected from the upper layer. The assays were performed using the ELISA kit (Abbkine) in accordance with the manufacturer’s instructions. The GF standards of known concentrations served as indirect reference controls for the determination of the levels of GFs present in the samples under investigation. Each sample was subjected to triplicate analysis, and the mean concentration was calculated.

### 2.7 Effect of H C-S/PRP on the proliferation of HUVECs

The HUVECs were thawed, resuspended, and cultured. Passaging was performed when the cells reached approximately 80% confluence. Subsequently, the cultured HUVECs were digested in order to obtain a suspension, and the cell counts were performed using a hemocytometer. Aliquots of 5 × 10⁴ cells were seeded on equal-sized H C-S/PRP scaffolds, H C-S scaffolds, or N C-S scaffolds. The scaffolds were disinfected using ultraviolet radiation. Following a 30-min incubation period in a constant temperature incubator to permit cellular attachment, a complete culture medium was introduced. MTT assays were conducted on days 3, 5, and 7 of the culture period. Simply put, MTT solution is added to the test sample, and then DMSO is used to dissolve the generated formazan in the cells. Transfer the color developed solution to a 96 well plate. The optical density (OD) values were determined at a wavelength of 490 nm. The cells and materials in the co-culture state were extracted concurrently and stored overnight in a −80°C freezer, followed by scanning electron microscope (SEM). Moreover, the proliferative effects of H C-S/PRP on HUVECs were investigated at the gene level using reverse transcription–quantitative polymerase chain reaction (RT-qPCR). On day 7, total RNA was extracted from HUVECs grown on H C-S/PRP scaffolds. In brief, 1 mL NAiso plus (TAKARA) was added to each sample and allowed to stand for 5 min at room temperature. The purified RNA was then extracted through the following steps: phase separation, RNA precipitation, washing and dissolution. The quantity of RNA was ascertained through the utilisation of a Nanodrop 2000. Subsequently, cDNA was synthesised using a reverse transcription kit (Beyotime). The resulting DNA was amplified using a Step-One-Plus real-time PCR system. The primers employed in this experiment included those for cyclin D1, cyclin D3, p53, c-Myc, Bcl-2 and GAPDH. The primer sequences are presented in Table 1. The PCR parameters were set as follows: initial denaturation at 95°C for 30 s, followed by denaturation at 95°C for 5 s, annealing and extension temperature at 60°C for 30 s, for a total of 40 cycles. The relative gene expression levels were calculated using the 2^−ΔΔCT^ method, with GAPDH as the reference gene.

**TABLE 1:** Displays primer sequences used in PCR experiments.

Gene	Sequences (5′–3′)
cyclin D1	F: GCG​AGC​CAC​ACG​TCA​AAC​TA
	R: TCC​GTT​GAT​AGA​CAC​AAC​TCC​TC
cyclin D3	F: CTG​TAA​CAT​CGA​AAC​TGG​GGA​AA
	R: CCA​TAG​CTG​AAC​TCA​AAA​CCA​CC
p53	F: CTG​CCG​TCC​GAT​TGA​GAC​C
	R: CCC​CTC​CTT​GTA​CCA​CTG​TC
c-Myc	F: CTG​CAA​CAG​ACT​TCC​ATC​CAG
	R: AGT​GCT​ATT​GAC​AGG​TCT​GTT​GG
Bcl-2	F: CCC​TCA​CAC​TCT​GAT​CAT​CTT​CT
	R: GCT​ACG​ACC​AGG​GCT​ACA​G
GAPDH	F: AGA​AGG​CTG​GGG​CTC​ATT​T
	R: GGT​GCT​AAG​CTG​TTG​GTG​GT

### 2.8 Constructing a bone-exposed wound model in porcine

The ethical guidelines for animal experimentation were rigorously adhered to throughout the course of this study, and all protocols were duly approved by the Ethics Committee of the Medical Department of Yangtze University (approval number: 202401005). Ten Bama pigs were anaesthetised with continuous intratracheal isoflurane and positioned on the operating table in the prone position. The heads of the subjects were immobilised and the surrounding hair was removed. The surgical area was sterilised with iodophor. In a sterile environment, two circular bone-exposed wounds, measuring 2 cm in diameter and preserving the periosteum, were created above the orbit using an 11-blade scalpel, with a 3 cm gap between them. The scaffolds were disinfected with ethylene oxide. The scaffolds were trimmed to fit the craniotomies and secured with 3–0 Ethicon sutures. The animals were divided into five groups: the control group, the PRP group, the N C-S group, the H C-S group and the H C-S/PRP group. The surgical wounds were dressed with sterile gauze, compressed and tightly bandaged in accordance with standard surgical practice. Once the anaesthetic had worn off, the pigs were transferred to individual cages for recovery. Post-operative analgesia and antibiotics were administered for a period of 3 days.

### 2.9 Gross observation and quantification of wound size

During the experimental period, photographic documentation was conducted on days 0, 10, and 30 to evaluate the status of the wounds, noting any indications of inflammation, infection, or exudate. Subsequently, sterile medical paper was applied to the wounds in order to create a visible impression, which was then analysed using ImageJ software to accurately measure the wound area.

### 2.10 Histological staining and scoring

Skin punch biopsies, with a diameter of 3 mm, were taken from the centre of the wound on days 10, 20 and 30. Subsequently, the specimens were fixed in 4% paraformaldehyde (Aladdin) for 1 h prior to undergoing a dehydration and paraffin embedding process. The subsequent step was the performance of a haematoxylin and eosin (HE) staining procedure, which was conducted for the purpose of histological analysis. The evaluation of the inflammatory response, vascular regeneration and epithelialisation at the wound site was conducted using histological scoring methods based on previous literature (de Moura Estevao et al., 2019). Furthermore, immunohistochemical staining for CD31 was conducted. In short, following dewaxing of the paraffin sections, antigen retrieval was conducted using citrate buffer (pH 6.0). Additionally, endogenous peroxidase activity was inactivated by treating each section with 3% H₂O₂ and incubating for approximately 10 min at room temperature. The primary antibody, CD31 (1:100 dilution), was added and incubated overnight at 4°C in a refrigerator. A secondary antibody was employed to specifically bind to the corresponding primary antibody. Subsequently, the nuclei in the samples were stained with haematoxylin. The samples were then subjected to a process of closed fixation, which involved the use of neutral gum and the subsequent addition of a coverslip.

### 2.11 Statistical analysis

All experimental data were collected with precision and objectivity, thus ensuring the reliability of our findings. The statistical analysis of the differences between the two groups was conducted using unpaired t-tests, while comparisons between multiple groups were performed using one-way ANOVA. All statistical calculations were performed using the SPSS 19 software. The results are expressed as mean ± standard deviation (SD), and a P-value of less than 0.05 is considered statistically significant, indicating a meaningful difference in the observations.

## 3 Results

### 3.1 Synthesis of H C-S and H C-S/PRP and their physical properties

**FIGURE 2:**
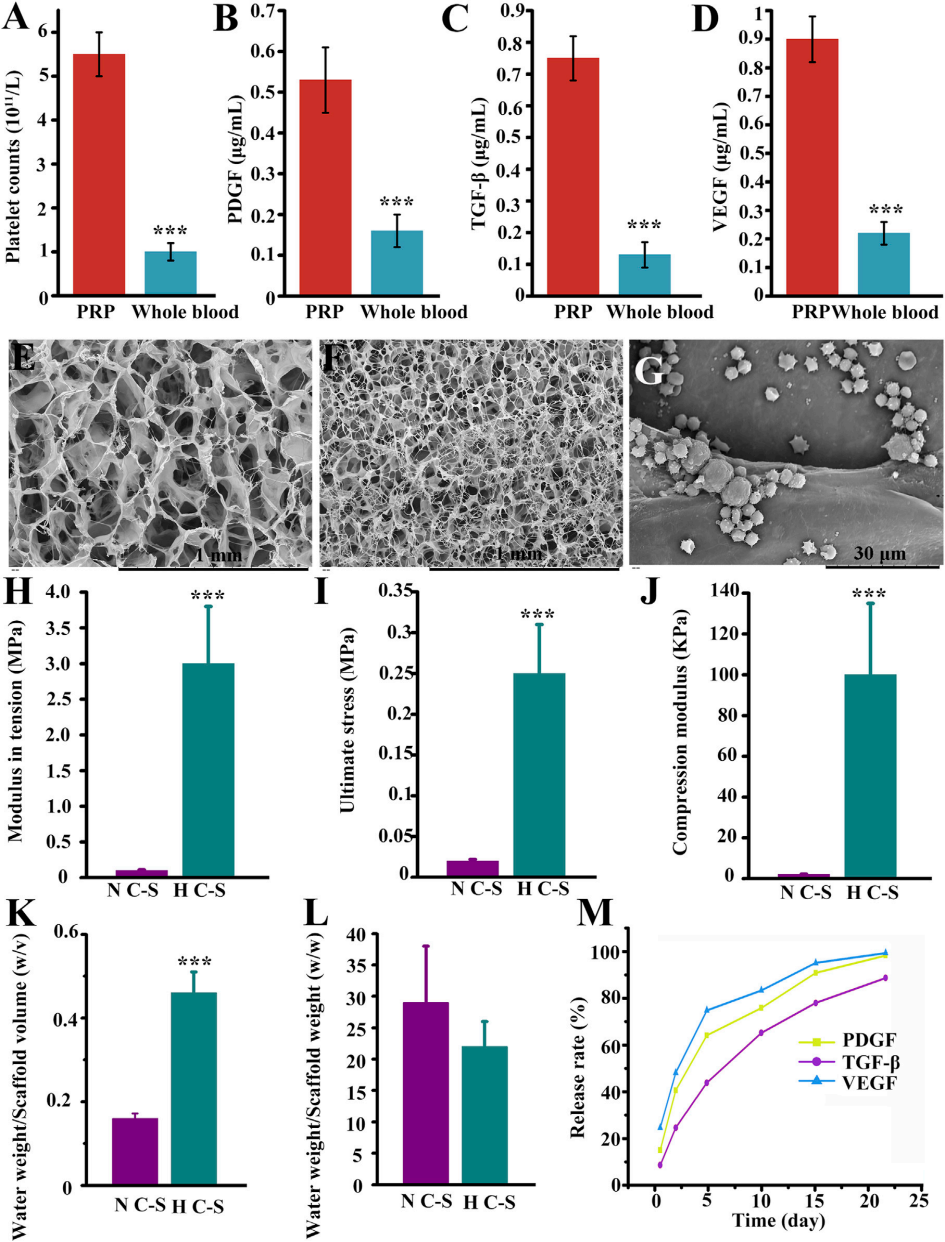
Synthesis and biological properties of H C-S/PRP. **(A)** Comparison of platelet content in PRP versus whole blood. **(B)** Comparison of PDGF content in PRP versus whole blood. **(C)** TGF-β in PRP versus whole blood. **(D)** Comparison of VEGF content in PRP versus whole blood. N C-S **(E)**, H C-S **(F)** and H C-S/PRP **(G)** SEM images. **(H)** Tensile modulus of H C-S scaffold. **(I)** Maximum tensile value of the H C-S scaffold. **(J)** Compressive modulus of the H C-S scaffold. **(K, L)** Water absorption property of H C-S. **(M)** Sustained release effect of PDGF, TGF-β and VEGF in H C-S scaffold. *** represents *p* < 0.001 compared to control.

The platelet concentration in the obtained PRP shown in Figure 2A was (5.5 ± 0.4) × 10^11^ cells/L, which was more than five times that of whole blood (0.98 ± 0.1) × 10^11^ cells/L, with a P-value of less than 0.000, and the difference was statistically significant. As illustrated in Figures 2B–D, the experimental results indicated that the concentrations of PDGF, TGF-β and VEGF in PRP were 0.53 ± 0.09 μg/mL, 0.75 ± 0.1 μg/mL, and 0.89 ± 0.1 μg/mL, whereas the concentrations of PDGF, TGF-β, and VEGF in whole blood were 0.17 ± 0.05 μg/mL, 0.12 ± 0.03 μg/mL, and 0.23 ± 0.04 μg/mL, respectively, with P values less than 0.000, and the differences were all statistically significant. The results indicated that the PRP had a high concentration of platelets and a high concentration of PDGF, TGF-β and VEGF. Figures 2E, F revealed the SEM images of N C-S and H C-S, the scaffolds all presented relatively uniform pores, the diameter of the pores in H C-S was smaller than that of N C-S, the diameter of the pores in N C-S was about 80–150 μm, and that in H C-S was about 30–60 μm. Figure 2G illustrated that the H C-S scaffold was adhered with spherical “mine-like” platelet particles with good size uniformity. Figure 2H displayed that the tensile modulus of H C-S was significantly higher than that of N C-S, almost 30 times higher. Figure 2I illustrated the ultimate stress values when the scaffold was fractured. Figure 2J demonstrated that the compressive modulus of H C-S was significantly higher than that of N C-S. Figures 2K, L indicated the water absorption of H C-S and N C-S at the 24 h of immersion in culture solution. As shown in Figure 2K, the water uptake of H C-S was significantly higher than that of N C-S at the same volume. However, the results presented in Figure 2L were not statistically significantly different when compared to the water uptake of N C-S, despite a decrease in water uptake of H C-S at the same weight. The release rates of 3 GFs, PDGF, TGF-β and VEGF, were measured on days 0.5, 2, 5, 10, 15, and 22, respectively, as shown in Figure 2M. The results demonstrated that the 3 GFs, PDGF, TGF-β and VEGF, were released at a slow rate. Both PDGF and VEGF were released on day 22, with a release rate close to 100%. TGF-β was released more slowly, with a release rate of approximately 90% on day 22.

### 3.2 Growth of HUVECs on HC-S/PRP scaffold

**FIGURE 3:**
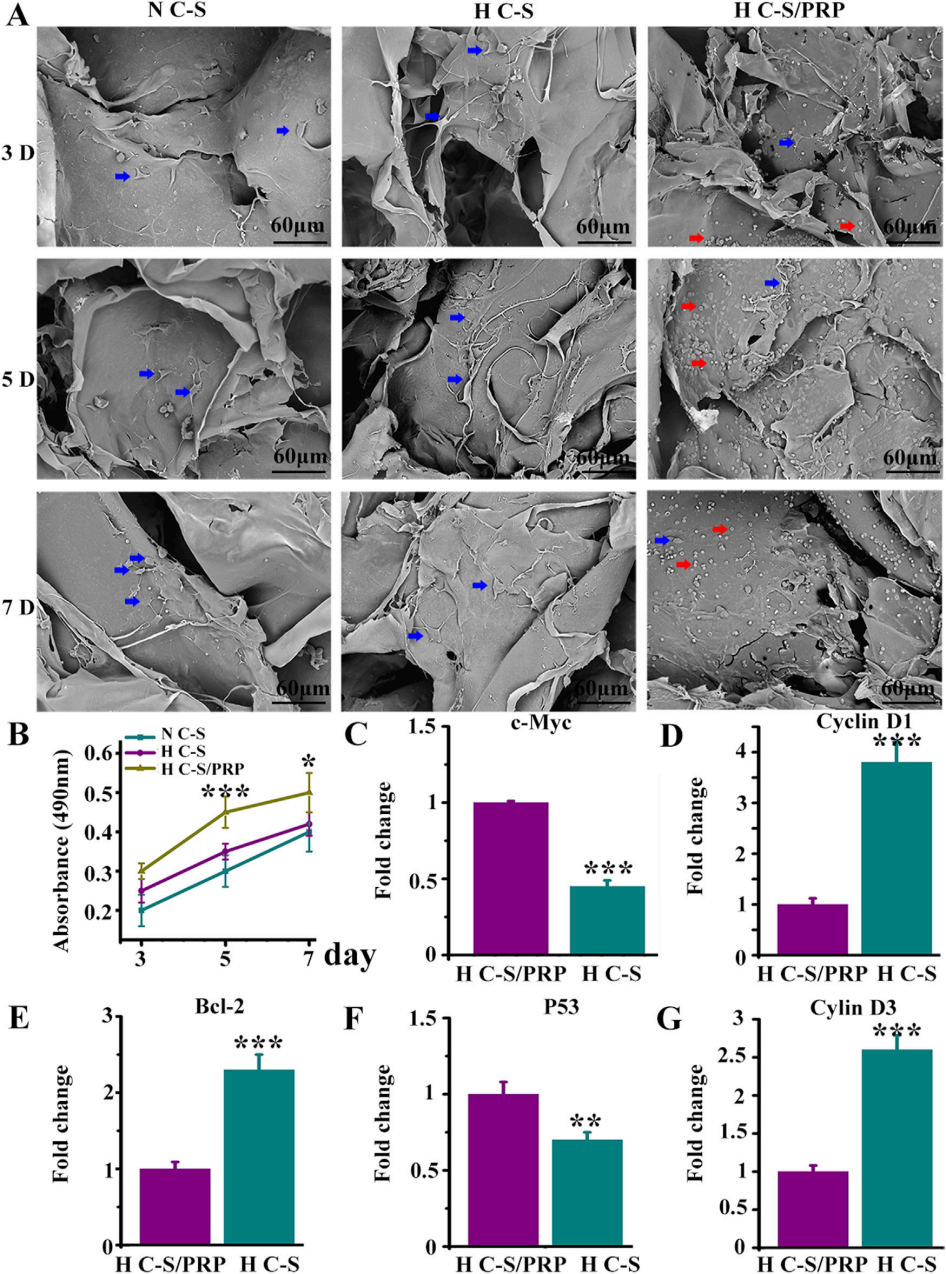
Growth of HUVECs on HC-S/PRP scaffold. **(A)** SEM image with red arrows indicating platelets and blue arrows indicating HUVECs. **(B)** Results of MTT experiments. **(C–G)** Expression of genes c-Myc, P53, Bcl-2, cyclin D3 and cyclin D1. ** represents *p* < 0.01 compared with control. *** represents *p* < 0.001 compared with control.

Figure 3A presented the growth of HUVECs on H C-S/PRP scaffold by SEM, which displayed good growth and normal morphology. The number of cells gradually increased with time, indicating that HUVECs were actively growing on the scaffold and that the scaffold was biocompatible. Figure 3B indicated that on days 3, 5 and 7, the H-C-S/PRP group promoted the proliferation of HUVECs more than the H-C-S and N-C-S groups, and the difference was statistically significant with a P-value of less than 0.05. The results of Figures 3C–G illustrated that the H C-S/PRP group significantly suppressed the expression of pro-apoptosis related genes c-Myc and P53. And significantly promoted the expression of inhibition of apoptosis-related gene Bcl-2 and pro-proliferation-related genes cyclin D3 and cyclin D1. The difference was statistically significant, P less than 0.05.

### 3.3 Gross observation of wound healing of bone-exposed in porcine

**FIGURE 4:**
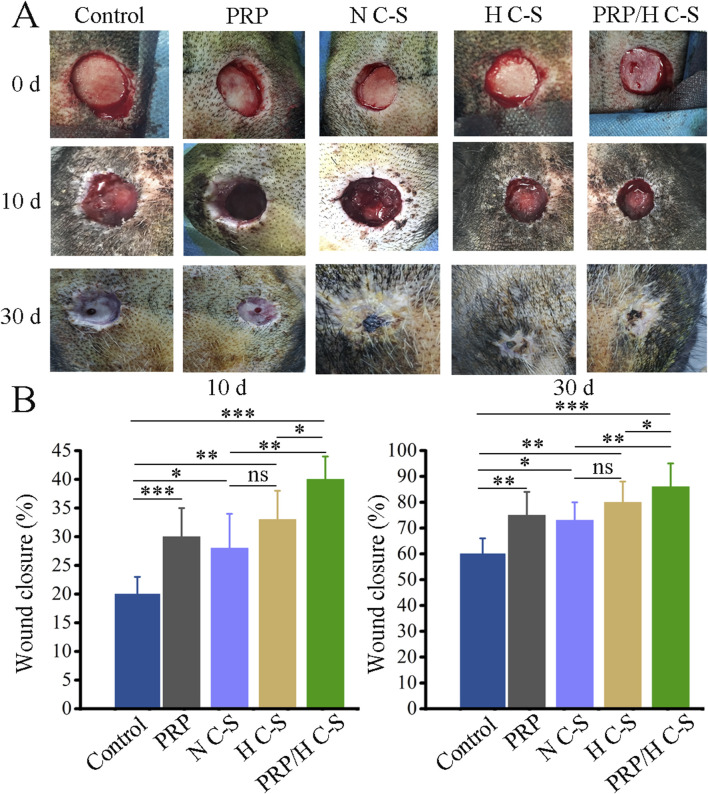
H C-S/PRP promotes wound healing of bone-exposed in porcine. **(A)** Wound healing at day 0, day 10 and day 30. **(B)** Quantification of wound healing rate at day 0, day 10 and day 30. * represents *p* < 0.01 compared to control. ** represents *p* < 0.01 compared with control. *** represents *p* < 0.001 compared with control.

Wound healing was counted on the day of surgery, 10th postoperative day and 30th postoperative day as illustrated in Figure 4A. The wounds in all groups healed gradually over time. However, it was observed that HC-S/PRP wounds healed the fastest, followed by H C-S and PRP, which were significantly faster than the control group. Figure 4B Quantitative statistics showed that at postoperative days 10 and 30, the wound healing rate was 46% ± 5.78% and 86% ± 4.32% in the H C-S/PRP group and 33% ± 7.34% and 80% ± 4.83% in the H C-S group, respectively. In addition, H C-S/PRP promoted wound healing significantly more than the N C-S group. The difference was statistically significant. Both quantitative and qualitative results clearly demonstrated that the H C-S/PRP group was more effective in promoting wound healing.

### 3.4 HE staining and scoring results

**FIGURE 5:**
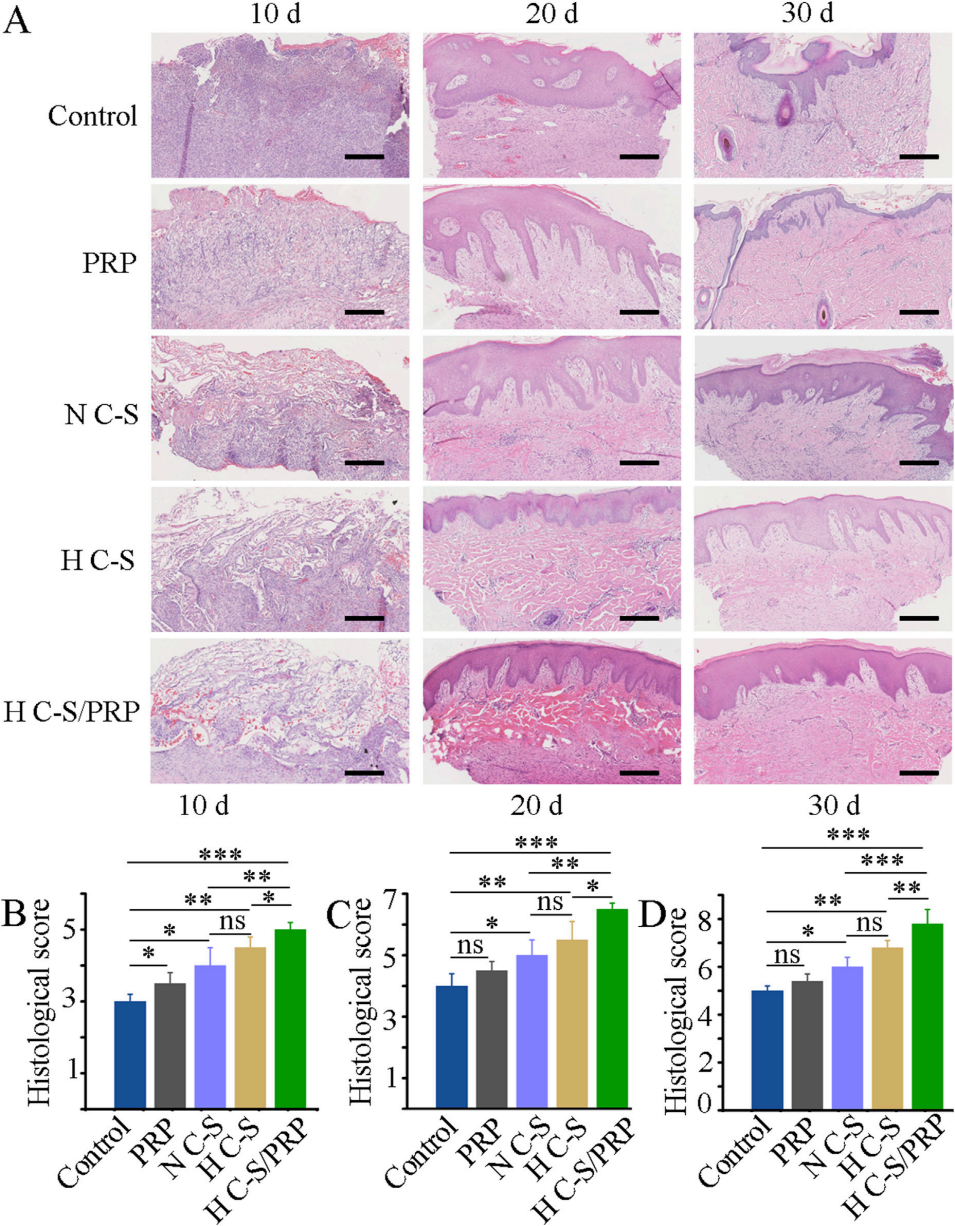
HE staining and scoring. **(A)** HE staining results of tissue at the wound site on days 10, 20 and 30. **(B)** Histological score of wound healing on day 10. **(C)** Histological score of wound healing on day 20. **(D)** Histological scores of wound healing on day 30. Scale bar is 2 mm. * represents *p* < 0.01 compared to control. ** represents *p* < 0.01 compared with control. *** represents *p* < 0.001 compared with control.

The results of HE staining indicated that, in general, there was a gradual increase in the regeneration of granulation tissue at the wound site and a gradual increase in collagen and epidermal tissue in all groups over time. Figure 5A illustrated that the H C-S/PRP group had more pronounced epithelial regeneration and collagen deposition compared to the other groups. Figure 5B demonstrated that on day 10 the wound histological score was significantly higher in the H C-S/PRP group compared to the other groups. Similarly, on days 20 and 30 as illustrated in Figures 5C, D, the wound histological scores revealed that the H C-S/PRP group was the most effective in promoting wound healing.

### 3.5 Results of CD31 immunohistochemical staining

**FIGURE 6:**
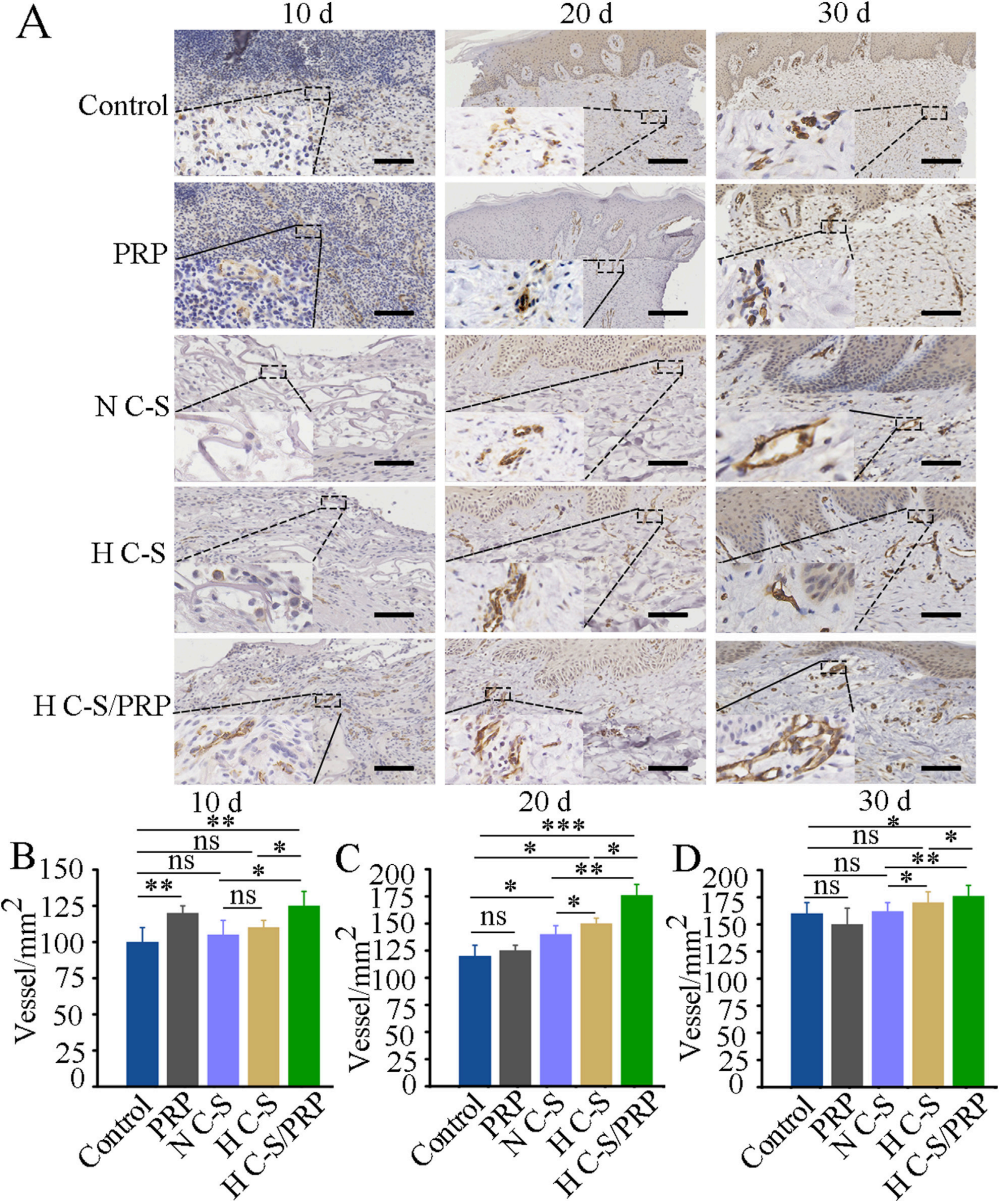
CD31 immunohistochemical staining results. **(A)** CD31 staining results of wound site tissues at day 10, day 20 and day 30, respectively. The lower left corner shows a partial enlargement of the image by 3.5 times. **(B)** Quantitative analysis of angiogenesis at the wound site at day 10. **(C)** Quantitative analysis of angiogenesis at the wound site on day 20. **(D)** Quantitative analysis of angiogenesis at the wound site on day 30. Scale bars are 2 mm. * represents *p* < 0.01 compared to control. ** represents *p* < 0.01 compared to control. *** represents *p* < 0.001 compared to control.

Vascular regeneration during wound healing was further investigated. As illustrated in Figure 6A, blood vessel regeneration was more pronounced in the HC-S/PRP group than in the control group on days 10, 20 and 30. Figure 6B demonstrated that the number of blood vessels at the wound site on day 10 was significantly higher in the H C-S/PRP group than in the control group. Similarly, as indicated in Figures 5C, D, the number of blood vessels at the wound site was significantly higher in the H C-S/PRP group compared to the control group on days 20 and 30. In addition, H C-S/PRP promoted the number of blood vessels significantly more than the N C-S group and the H C-S group.

## 4 Discussion

Bone-exposed wounds represent a severe form of skin and soft tissue deficiency that necessitates the use of high-performance wound dressings. It is essential that these dressings possess not only adequate mechanical strength but also meet the biochemical conditions required for optimal wound healing. In this study, we sought to enhance the mechanical strength of the scaffold material by adjusting the concentration of collagen and to utilise the properties of PRP, which is rich in GFs that promote tissue and vascular regeneration, to develop a treatment protocol suitable for the healing of bone-exposed wounds. To the best of our knowledge, there are relatively few studies that have employed large animal models, such as porcine, for the investigation of bone-exposed wounds. However, the similarity between porcine and human skin lends additional credibility to our results and provides valuable insights for clinical applications.

A crucial aspect of the utilisation of scaffold materials in the treatment of bone-exposed wounds is the provision of an adequate mechanical environment until such time as the formation of granulation tissue and epidermis at the wound site is sufficiently advanced (Freedman et al., 2023). To ascertain whether elevated collagen concentrations could enhance the mechanical attributes of the scaffolds, tensile and compressive loading tests were conducted. The results demonstrated that the tensile and compressive modulus of elasticity of the H C-S scaffolds were markedly higher than those of the scaffolds with general concentrations, thereby providing enhanced mechanical protection to the wound. Through this experiment, we established that increasing the collagen concentration within the scaffold could potentially augment its mechanical strength. This approach of tailoring the mechanical strength of the scaffold is likely to facilitate tissue repair in disparate mechanical environments.

The results of this experiment suggest that scaffolds composed of H C-S facilitate the orderly release of GFs from PRP, thereby demonstrating a sustained release effect. Previous research has indicated that the gradual release of GFs is conducive to the promotion of wound healing (Qian et al., 2020). PRP is a source of numerous GFs and cytokines. The presence of PDGF, VEGF and TGF-β was confirmed in the PRP obtained in this study. PDGF and VEGF are essential for the process of angiogenesis. PDGF has been demonstrated to enhance the survival, proliferation and migration of endothelial cells, thereby playing a role in the promotion of angiogenesis (Zhang et al., 2022). Recombinant human PDGF has demonstrated efficacy in promoting wound healing (Gao et al., 2021). VEGF has been demonstrated to promote mitosis and anti-apoptotic activity in endothelial cells, increase vascular permeability and facilitate endothelial cell migration, thereby promoting angiogenesis (Perez-Gutierrez and Ferrara, 2023). These findings are in accordance with the experimental results, which revealed that the incorporation of PRP into H C-S scaffolds markedly enhanced endothelial cell proliferation. Prior experimental studies have indicated that PRP promotes the proliferation of human keratinocytes (HaCaT) cells by regulating cell cycle progression (Misiura et al., 2021). To further investigate whether PRP promotes endothelial cell proliferation through cell cycle modulation, the expression of cell cycle-related genes, including c-Myc, P53, Bcl-2, cyclin D1, and cyclin D3, was analysed using RT-qPCR in this experiment. The expression of apoptosis-related genes, including c-Myc and P53, was found to be inhibited in the H C-S/PRP group, while the expression of the anti-apoptotic gene Bcl-2 was increased and that of cell cycle activators, including cyclin D1 and cyclin D3, was promoted. These findings suggest that PRP may enhance endothelial cell proliferation by influencing the cell cycle.

The process of wound healing is typically divided into four distinct phases: hemostasis, inflammation, proliferation, and remodeling. GFs are known to play a pivotal role in all of these phases (Li et al., 2024). Of these, TGF-β is of particular importance throughout the entirety of the wound healing process (Bonnici et al., 2023), with a particular influence on the proliferation and remodeling phases. During these stages, it facilitates the formation of granulation tissue, angiogenesis, and epithelial regeneration (Li et al., 2017). In this experiment, a variety of materials were applied to cranial bone exposure wounds of Bama mini-pigs, including N C-S, H C-S, H C-S/PRP and PRP alone. It was observed that the H C-S/PRP group exhibited significantly accelerated wound healing. Furthermore, histological staining experiments demonstrated that the regeneration of granulation tissue and epidermis was markedly enhanced in the H C-S/PRP group, which also received higher histological scores. This improvement is likely to be associated with the release of GFs from PRP.

The transportation of nutrients and promotion of cellular metabolism are essential processes during the wound healing process, and the role of blood vessels in this process cannot be underestimated. Effective vascular regeneration is a crucial factor in the process of wound healing, exerting a direct influence on the quality and speed of the healing process (Yu et al., 2024). It has been demonstrated that angiogenesis initially occurs through the proliferation and migration of endothelial cells, which then give rise to the formation of nascent vascular sprouts. Subsequently, these sprouts develop lumens under the influence of blood flow and recruit pericytes from the stroma to adhere to the new vessels, thereby completing the maturation of the neovasculature (DiPietro, 2016). The results of the experimental study demonstrated that the H C-S/PRP group, which was enriched with GFs such as PDGF, VEGF and TGF-β, significantly promoted the proliferation of vascular endothelial cells, thereby facilitating vascular regeneration. Interestingly, the *in vivo* experiments confirmed this effect. The CD31 immunohistochemistry results indicated that the H C-S/PRP group significantly enhanced vascular regeneration at the wound site.

The preclinical success of our study using a porcine model for bone-exposed wound healing with H C-S/PRP scaffolds paves the way for potential clinical translation. The mechanical and biochemical properties of our scaffold, which have been optimized in a large animal model, suggest that it could be particularly beneficial in human wound care scenarios requiring both structural support and a conducive environment for tissue regeneration. For instance, our scaffold may be ideally suited for patients with extensive burn injuries, chronic wounds, or those undergoing reconstructive surgeries where traditional grafts are either insufficient or unavailable. However, the transition from preclinical to clinical use is not without challenges. One of the primary considerations is the scalability of our production process to meet clinical demands while maintaining the quality and integrity of the scaffolds. Additionally, the porcine model, despite its similarities to human skin, may not fully replicate the complexity of the human wound healing process. Therefore, further research is needed to tailor our findings to the nuances of human biology. Another anticipated challenge is navigating the regulatory landscape. The approval process for new medical devices and treatments is rigorous and requires extensive safety and efficacy testing. We must also consider the economic implications, including the cost of production and the potential for reimbursement by healthcare systems, which could influence the widespread adoption of H C-S/PRP scaffolds. It is important to acknowledge the limitations inherent in our study. The use of a porcine model, while providing valuable insights, may not perfectly translate to human wound healing due to species-specific differences in physiology and immune response. The duration of our study may not fully capture the long-term performance and safety of the H C-S/PRP scaffolds. Additionally, our study focused on a specific type of wound in a controlled setting, which may limit the generalizability of our findings to other wound types or more variable clinical environments. Furthermore, while our study demonstrated the potential for enhanced vascular and tissue regeneration, the specific mechanisms by which the scaffold and PRP interact with the human body’s complex wound healing processes require further elucidation. This includes understanding the scaffold’s interaction with the immune system and its potential impact on wound infection rates and scarring. Addressing these limitations will be crucial for the future development of our scaffold. This includes extending the study duration to assess long-term outcomes, conducting additional trials with diverse wound types, and refining our understanding of the scaffold’s mechanistic interactions with human biology. Overcoming these barriers will be essential to realize the full potential of H C-S/PRP scaffolds in clinical practice.

## 5 Conclusion

The synthesis of the H C-S scaffold was successfully completed and demonstrated to exhibit markedly enhanced mechanical characteristics. Furthermore, the combination of the H C-S scaffold with PRP was observed to markedly accelerate the healing of bone-exposed wounds in porcine models. This improvement is associated with the scaffold’s capacity to release a range of GFs in a structured manner, thereby facilitating tissue and vascular regeneration.

## Data Availability

The original contributions presented in the study are included in the article/Supplementary Material, further inquiries can be directed to the corresponding author.

## References

[B1] Bonnici L. Suleiman S. Schembri-Wismayer P. Cassar A. (2023). Targeting signalling pathways in chronic wound healing. Int. J. Mol. Sci. 25 (1), 50. 10.3390/ijms25010050 38203220 PMC10779022

[B2] Cao X. Lin X. Li N. Zhao X. Zhou M. Zhao Y. (2023). Animal tissue-derived biomaterials for promoting wound healing. Mater Horiz. 10 (9), 3237–3256. 10.1039/d3mh00411b 37278612

[B3] Darwish Y. G. Gharbia M. Sobhi A. M. Aborady A. F. (2024). The benefits of using platelet-rich plasma with dermal substitutes for extremity posttraumatic skin defects: a short-term outcome. Plast. Reconstr. Surg. Glob. Open 12 (1), e5492. 10.1097/GOX.0000000000005492 38288418 PMC10817021

[B4] de Moura Estevao L. R. Cassini-Vieira P. Leite A. G. B. de Carvalho Bulhoes A. A. V. da Silva Barcelos L. Evencio-Neto J. (2019). Morphological evaluation of wound healing events in the excisional wound healing model in rats. Bio Protoc. 9 (13), e3285. 10.21769/BioProtoc.3285 PMC7854106 33654800

[B5] DiPietro L. A. (2016). Angiogenesis and wound repair: when enough is enough. J. Leukoc. Biol. 100 (5), 979–984. 10.1189/jlb.4MR0316-102R 27406995 PMC6608066

[B6] Everts P. A. van Erp A. DeSimone A. Cohen D. S. Gardner R. D. (2021). Platelet rich plasma in orthopedic surgical medicine. Platelets 32 (2), 163–174. 10.1080/09537104.2020.1869717 33400591

[B7] Fani N. Moradi M. Zavari R. Parvizpour F. Soltani A. Arabpour Z. (2024). Current advances in wound healing and regenerative medicine. Curr. Stem Cell. Res. Ther. 19 (3), 277–291. 10.2174/1574888X18666230301140659 36856176

[B8] Freedman B. R. Hwang C. Talbot S. Hibler B. Matoori S. Mooney D. J. (2023). Breakthrough treatments for accelerated wound healing. Sci. Adv. 9 (20), eade7007. 10.1126/sciadv.ade7007 37196080 PMC10191440

[B9] Gao S. Y. Lin R. B. Huang S. H. Liang Y. J. Li X. Zhang S. E. (2021). PDGF-BB exhibited therapeutic effects on rat model of bisphosphonate-related osteonecrosis of the jaw by enhancing angiogenesis and osteogenesis. Bone 144, 115117. 10.1016/j.bone.2019.115117 31676407

[B10] Grzelak A. Hnydka A. Higuchi J. Michalak A. Tarczynska M. Gaweda K. (2024). Recent achievements in the development of biomaterials improved with platelet concentrates for soft and hard tissue engineering applications. Int. J. Mol. Sci. 25 (3), 1525. 10.3390/ijms25031525 38338805 PMC10855389

[B11] Guo Y. Ding S. Shang C. Zhang C. Li M. Zhang Q. (2024). Multifunctional PtCuTe nanosheets with strong ROS scavenging and ROS-independent antibacterial properties promote diabetic wound healing. Adv. Mater 36 (8), e2306292. 10.1002/adma.202306292 37723937

[B12] Harrison P. Subcommittee on Platelet P. Andia I. Burnouf T. Dohan Ehrenfest D. Everts P. (2018). The use of platelets in regenerative medicine and proposal for a new classification system: guidance from the SSC of the ISTH. J. Thromb. Haemost. 16 (9), 1895–1900. 10.1111/jth.14223 30099839

[B13] Haxaire C. Liebel F. Portocarrero Huang G. Chen S. Knapp E. Idkowiak-Baldys J. (2023). Effect of L-4-Thiazolylalanine (Protinol) on skin barrier strength and skin protection. Int. J. Cosmet. Sci. 45 (6), 725–738. 10.1111/ics.12881 37402136

[B14] Kahn S. A. Carter J. E. Wilde S. Chamberlain A. Walsh T. P. Sparks J. A. (2024). Autologous skin cell suspension for full-thickness skin defect reconstruction: current evidence and Health economic expectations. Adv. Ther. 41 (3), 891–900. 10.1007/s12325-023-02777-7 38253788 PMC10879381

[B15] Li X. Zhang D. Yu Y. Wang L. Zhao M. (2024). Umbilical cord-derived mesenchymal stem cell secretome promotes skin regeneration and rejuvenation: from mechanism to therapeutics. Cell. Prolif. 57 (4), e13586. 10.1111/cpr.13586 38148579 PMC10984109

[B16] Li Y. Zhang J. Yue J. Gou X. Wu X. (2017). Epidermal stem cells in skin wound healing. Adv. Wound Care (New Rochelle) 6 (9), 297–307. 10.1089/wound.2017.0728 28894637 PMC5592843

[B17] Liang W. H. Kienitz B. L. Penick K. J. Welter J. F. Zawodzinski T. A. Baskaran H. (2010). Concentrated collagen-chondroitin sulfate scaffolds for tissue engineering applications. J. Biomed. Mater Res. A 94 (4), 1050–1060. 10.1002/jbm.a.32774 20694972 PMC2922023

[B18] Misiura M. Guszczyn T. Oscilowska I. Baszanowska W. Palka J. Miltyk W. (2021). Platelet-rich plasma promotes the proliferation of human keratinocytes via a progression of the cell cycle. A role of prolidase. Int. J. Mol. Sci. 22 (2), 936. 10.3390/ijms22020936 33477820 PMC7832888

[B19] Motsoene F. Abrahamse H. Dhilip Kumar S. S. (2023). Multifunctional lipid-based nanoparticles for wound healing and antibacterial applications: a review. Adv. Colloid Interface Sci. 321, 103002. 10.1016/j.cis.2023.103002 37804662

[B20] Pena O. A. Martin P. (2024). Cellular and molecular mechanisms of skin wound healing. Nat. Rev. Mol. Cell. Biol. 25 (8), 599–616. 10.1038/s41580-024-00715-1 38528155

[B21] Perez-Gutierrez L. Ferrara N. (2023). Biology and therapeutic targeting of vascular endothelial growth factor A. Nat. Rev. Mol. Cell. Biol. 24 (11), 816–834. 10.1038/s41580-023-00631-w 37491579

[B22] Pineda-Cortel M. R. Suarez C. Cabrera J. T. Daya M. Bernardo-Bueno M. M. Vergara R. C. (2023). Biotherapeutic applications of platelet-rich plasma in regenerative medicine. Tissue Eng. Regen. Med. 20 (6), 811–828. 10.1007/s13770-023-00560-x 37651090 PMC10519926

[B23] Pixley J. N. Cook M. K. Singh R. Larrondo J. McMichael A. J. (2023). A comprehensive review of platelet-rich plasma for the treatment of dermatologic disorders. J. Dermatol. Treat. 34 (1), 2142035. 10.1080/09546634.2022.2142035 36318219

[B24] Qian Z. Wang H. Bai Y. Wang Y. Tao L. Wei Y. (2020). Improving chronic diabetic wound healing through an injectable and self-healing hydrogel with platelet-rich plasma release. ACS Appl. Mater Interfaces 12 (50), 55659–55674. 10.1021/acsami.0c17142 33327053

[B25] Rong H. Dong Y. Zhao J. Zhang X. Li S. Sun Y. (2023). Fetal milieu-simulating hyaluronic acid-dopamine-chondroitin sulfate hydrogel promoting angiogenesis and hair regeneration for wound healing. Int. J. Biol. Macromol. 248, 125739. 10.1016/j.ijbiomac.2023.125739 37423445

[B26] Schlottmann F. Strauss S. Ziesing S. Reineke C. Ipaktchi R. Weyand B. (2024). Organization of Hannover Skin Bank: sterile culture and procurement protocols for viable cryopreserved allogeneic skin grafts of living donors. Int. Wound J. 21 (1), e14374. 10.1111/iwj.14374 37675770 PMC10784195

[B27] Song H. Hao D. Zhou J. Farmer D. Wang A. (2024). Development of pro-angiogenic skin substitutes for wound healing. Wound Repair Regen. 32 (3), 208–216. 10.1111/wrr.13154 38308588

[B28] Wang T. W. Sun J. S. Wu H. C. Tsuang Y. H. Wang W. H. Lin F. H. (2006). The effect of gelatin-chondroitin sulfate-hyaluronic acid skin substitute on wound healing in SCID mice. Biomaterials 27 (33), 5689–5697. 10.1016/j.biomaterials.2006.07.024 16908060

[B29] Wang Y. Zhang Y. Li T. Shen K. Wang K. J. Tian C. (2023). Adipose mesenchymal stem cell derived exosomes promote keratinocytes and fibroblasts embedded in collagen/platelet-rich plasma scaffold and accelerate wound healing. Adv. Mater 35 (40), e2303642. 10.1002/adma.202303642 37342075

[B30] Wu S. Guo W. Li R. Zhang X. Qu W. (2022). Progress of platelet derivatives for cartilage tissue engineering. Front. Bioeng. Biotechnol. 10, 907356. 10.3389/fbioe.2022.907356 35782516 PMC9243565

[B31] Yu J. Gao Z. Han Q. Wang Z. Zhang W. Zhao J. (2024). Carboxymethyl chitosan-methacrylic acid gelatin hydrogel for wound healing and vascular regeneration. Biomed. Mater 19 (4), 045032. 10.1088/1748-605X/ad5482 38838692

[B32] Zhang Z. Warner K. A. Mantesso A. Nor J. E. (2022). PDGF-BB signaling via PDGFR-β regulates the maturation of blood vessels generated upon vasculogenic differentiation of dental pulp stem cells. Front. Cell. Dev. Biol. 10, 977725. 10.3389/fcell.2022.977725 36340037 PMC9627550

