# Supplementary figures and images for: Highly concentrated collagen/chondroitin sulfate scaffold with platelet-rich plasma promotes bone-exposed wound healing in porcine
Source: Front Bioeng Biotechnol. 2024 Sep 24;12:1441053. doi: 10.3389/fbioe.2024.1441053 (PMC11458455; doi:10.3389/fbioe.2024.1441053)

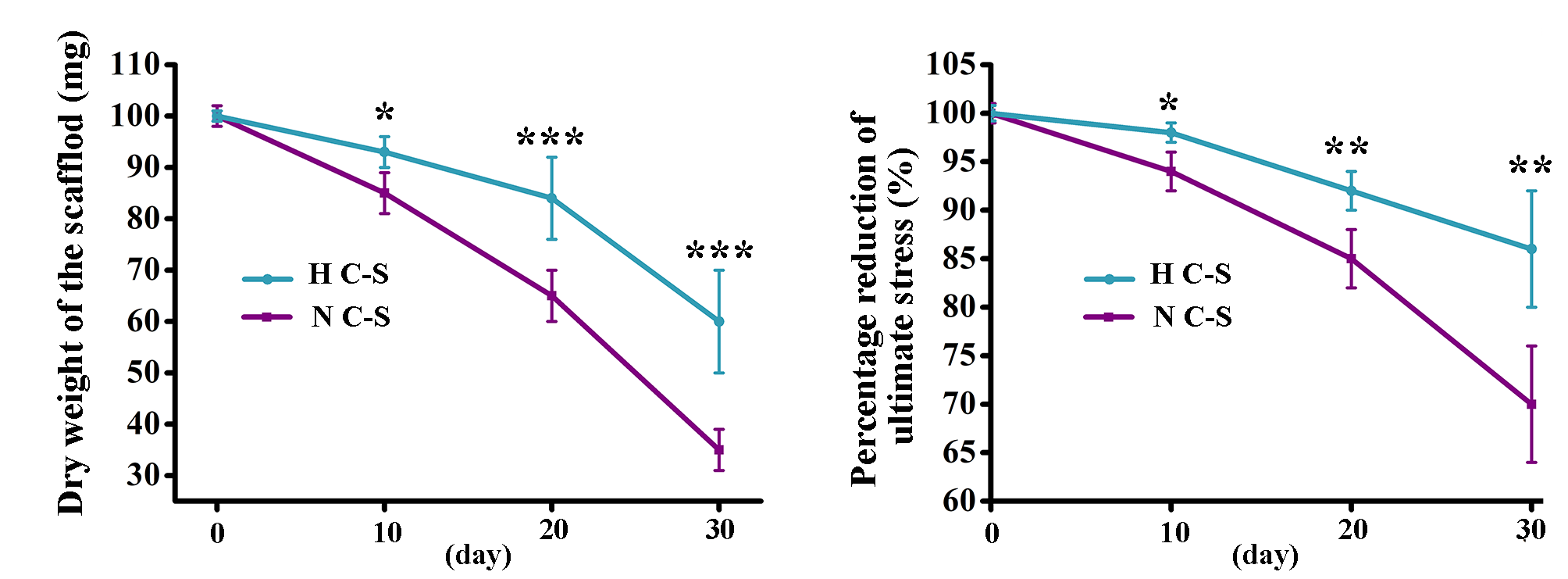

Supplement: Supplementary file 1 [file Image1.TIF]
