# Supplementary material for: Highly concentrated collagen/chondroitin sulfate scaffold with platelet-rich plasma promotes bone-exposed wound healing in porcine
Source: Front Bioeng Biotechnol. 2024 Sep 24;12:1441053. doi: 10.3389/fbioe.2024.1441053 (PMC11458455; doi:10.3389/fbioe.2024.1441053)
Supplement: Supplementary file 2 [file DataSheet1.docx]

In Figure S1A, high concentration collagen/chondroitin sulfate scaffolds degrade slower than normal concentration collagen/chondroitin sulfate scaffolds. In addition, in terms of mechanical properties, as indicated in Figure S1B, high concentration collagen/chondroitin sulfate scaffolds have smaller changes in ultimate stress values than normal concentration collagen/chondroitin sulfate scaffolds.

| 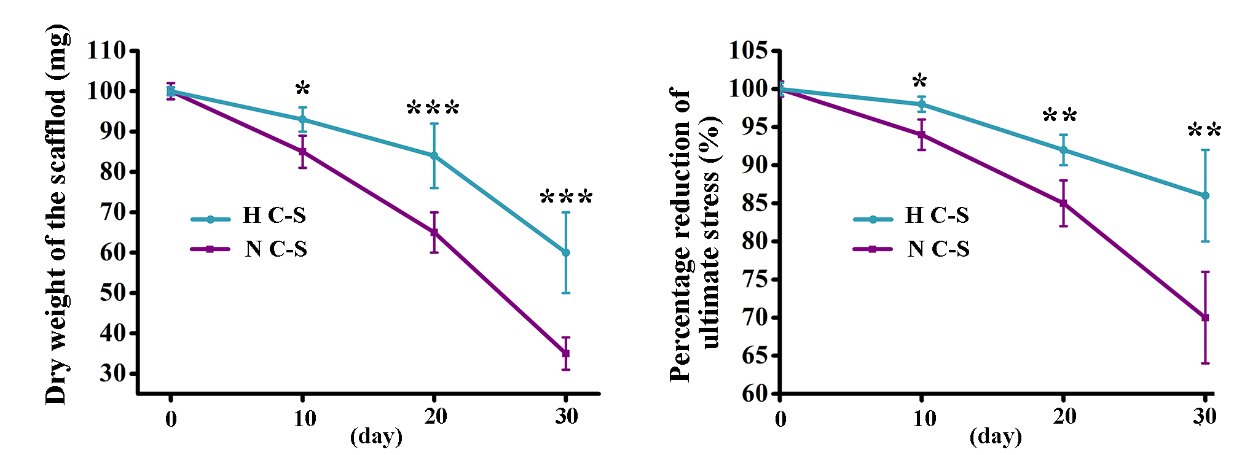 |
| --- |

Figure S1. The degradation rates and the mechanical properties of the scaffold. (A) The degradation rates of the scaffolds, (B) Changes in the mechanical properties of the scaffolds. * represents p<0.05 compared to control. ** represents p<0.01 compared with control. *** represents p<0.001 compared with control.
